# Concomitant acromioplasty is associated with enhanced structural healing, long-term durability, and functional outcomes in arthroscopic rotator cuff repair: a retrospective propensity score and causal machine-learning analysis

**DOI:** 10.3389/fbioe.2026.1840528

**Published:** 2026-10-07

**Authors:** Yucheng Song, Liang Zhao, Can Liu, Yijun Wang

**Affiliations:** 1 Shoulder Research Institute, Academy of Orthopedics Guangdong Province, Guangzhou, China; 2 Department of Shoulder and Elbow Surgery, Southern Medical University Third Hospital, Guangzhou, China

**Keywords:** acromion, arthroscopy, machine learning, propensity score, rotator cuff injuries

## Abstract

**Background:**

The clinical value of acromioplasty (AC) combined with arthroscopic rotator cuff repair (RCR) remains controversial. This study evaluates the associations and heterogeneity of treatment response for concomitant AC during arthroscopic RCR.

**Methods:**

We retrospectively analysed 734 patients (220 AC, 514 RCR-only). Propensity scores were estimated via a SuperLearner ensemble, followed by inverse probability of treatment weighting to balance covariates. Structural healing, functional outcomes (American Society of Shoulder and Elbow Surgeons and Constant–Murley Shoulder scores), and re-tear events were assessed at 12 months. Doubly robust machine-learning approaches (DR-learner, causal forest, Bayesian additive regression trees) were employed to estimate the average and individual treatment effects. Shapley Additive Explanations (SHAP) were used to improve model interpretability.

**Results:**

Acromioplasty was associated with improved structural healing (risk difference: −7.8%; 95% confidence interval [CI], −11.9% to −3.6%; number needed to treat [NNT] ≈ 13). Weighted Kaplan-Meier analysis showed significantly lower cumulative re-tear incidence at 60 months (AC: 45.9% vs. RCR-only: 80.0%; hazard ratio: 0.41; 95% CI: 0.33 to 0.51; p < 0.001). Functional scores were significantly higher with AC (ΔASES = 7.9; ΔConstant = 7.5; both p < 0.001), although these differences did not exceed established minimal clinically important difference thresholds. Considerable treatment heterogeneity was identified: patients with a high critical shoulder angle (CSA) (>35°) and older age (>62 years) showed the strongest associations with potential benefits. The SHAP analysis identified age, body mass index, tear size, and CSA as key factors influencing outcomes.

**Conclusion:**

Concomitant AC is associated with enhanced structural healing and long-term durability in arthroscopic RCR. Although functional scores were statistically higher in the AC group, the differences did not exceed established MCID thresholds, indicating limited clinical significance for functional outcomes. Significant treatment heterogeneity highlights the importance of individualised surgical strategies, particularly favouring patients with high CSAs and of older age.

## Introduction

Rotator cuff tears represent one of the most frequent shoulder disorders encountered by orthopaedic surgeons, significantly impacting patient quality of life and imposing substantial healthcare burdens globally (Testa et al., 2024; Toro et al., 2024). Arthroscopic rotator cuff repair (RCR) has become increasingly utilised and represents a preferred approach for many surgeons, demonstrating advantages over open repair in terms of reduced morbidity and quicker recovery, with comparable or favourable clinical outcomes reported in several studies (Darbandi et al., 2024; Gaudiani et al., 2024). However, structural healing following arthroscopic RCR remains a persistent clinical challenge, with re-tear rates reported as high as 20%–40%, particularly among older patients, in cases where larger tears have occurred and involve shoulders with anatomical risk factors (Gerber et al., 2018; Romeo et al., 2023).

Acromioplasty (AC), also termed subacromial decompression, has historically been proposed as an adjunctive procedure during arthroscopic RCR, aimed at reducing the extrinsic mechanical compression of repaired tendons and, theoretically, improving tendon healing (Caffard et al., 2023; Clark et al., 2025; Yang et al., 2020). Significant debate continues over its clinical efficacy and necessity, however, especially given recent randomised trials reporting equivocal or limited benefit in routine application (Hovland et al., 2025; Oladimeji et al., 2023; Yang et al., 2025). Oladimeji et al. conducted a landmark randomised placebo-controlled trial that questions the routine use of AC for impingement syndrome without cuff tears, triggering broader scepticism about its general application in shoulder surgery (Oladimeji et al., 2023).

Notably, recent biomechanical and imaging studies have highlighted critical shoulder angle (CSA), age, and tear size as potential predictors of poorer healing and higher re-tear risk (Oladimeji et al., 2023; Rancu et al., 2024; Ueda et al., 2024; Warner et al., 2025). Moore et al. identified an increased CSA as a biomechanical risk factor predisposing patients to RC pathology through increased lateral deltoid forces (Moore et al., 2025). Similarly, older age and a larger tear size have repeatedly emerged as important predictors of repair failure due to degenerative changes and impaired healing biology (Cohn et al., 2024). Despite this evidence, few studies have systematically examined how anatomical and patient-specific factors modify the effectiveness of AC.

Observational studies often suffer from confounding and selection bias, limiting causal interpretation. Advanced causal inference methods, such as propensity-score weighting and causal machine learning (ML) models, have emerged as robust analytical tools capable of addressing confounding and estimating patient-specific treatment effects (Gong et al., 2021; Javed et al., 2022; Wang et al., 2025). Methods like causal forest and Bayesian Additive Regression Trees (BART) offer powerful frameworks to estimate and interpret individual treatment-effect heterogeneity, supporting precision-medicine paradigms (Jain et al., 2025; Sinha et al., 2021; Suhre et al., 2020). Shapley Additive Explanations (SHAP) further provide interpretability, elucidating how individual clinical features influence model predictions (Jain et al., 2025).

Given the clinical uncertainty concerning concomitant AC during RCR, coupled with recent methodological advances in causal inference and predictive modelling, we aimed to determine the associations between AC and structural healing, functional outcomes and treatment-effect heterogeneity. This study addresses current gaps by combining robust causal methods with interpretable machine-learning analyses, providing empirical support for individualised decision-making and surgical strategy optimisation in arthroscopic shoulder surgery. This study addresses three primary research questions: (1) What is the association between concomitant AC and structural healing at 12 months after arthroscopic RCR? (2) Does concomitant AC show differential associations with outcomes across patient subgroups defined by CSA, age, and tear characteristics? (3) What patient-specific and anatomical factors most strongly influence the heterogeneity of treatment response? We hypothesised that concomitant AC would be associated with significantly improved clinical and structural outcomes in selected patient populations, particularly those with anatomical predispositions such as high CSA, older age, and larger tear dimensions. We expected that advanced causal inference methods would identify specific patient subgroups that would benefit most from concomitant AC.

## Methods

### Study design and patient selection

This is a retrospective cohort study of patients who underwent arthroscopic RCR at our institution between 2015 and 2024. We initially identified 1,217 consecutive cases from the electronic medical record, based on the billing code and description of the procedure. Patients were included if they underwent primary arthroscopic repair of a full-thickness supraspinatus or infraspinatus tear. Exclusion criteria included open or minimally invasive repair (n = 58), revision surgery (n = 44), isolated or partial subscapularis tears (n = 98), and surgery for concomitant fracture fixation or glenohumeral arthritis (n = 37). Other exclusion criteria included a lack of baseline magnetic resonance imaging (MRI) scans (n = 74), loss to follow-up within 12 months after surgery (n = 92), and incomplete demographic or patient-reported outcome (PRO) data (n = 80). To evaluate potential selection bias from exclusions, we compared baseline age, sex distribution, and tear size between the 172 excluded patients (92 lost to follow-up and 80 with incomplete data) and the 734 included patients; no clinically meaningful differences were observed (all SMD <0.10). After exclusions, 734 patients remained in the final included cohort, of whom 220 underwent concurrent AC (the AC group) and 514 underwent isolated RCR (the RCR-only group).

The decision to perform concomitant AC was made intraoperatively by the attending surgeon, based on specific clinical and anatomical criteria. Acromioplasty was performed for patients with one or more of the following indications: (1) the presence of a prominent anterolateral acromial spur, visualised arthroscopically; (2) CSA >35° on preoperative radiographs; (3) type II (curved) or type III (hooked) acromion morphology on outlet view radiographs; (4) evidence of subacromial impingement with mechanical contact between the acromion and RC during dynamic testing; and (5) an acromiohumeral distance <7 mm on anteroposterior (AP) radiographs. Patients without these anatomical risk factors typically underwent isolated RCR without AC.

Cases were retrospectively identified from our institutional database using Current Procedural Terminology (CPT) codes 29827 (arthroscopic RCR) and 29826 (arthroscopic subacromial decompression/AC). To ensure the accuracy of group assignment, we cross-referenced CPT coding with operative reports. All operative reports were manually reviewed to confirm whether AC had actually been performed, regardless of billing codes, to minimise misclassification. Patients were assigned to the AC group only if the operative report explicitly documented bursal excision, coracoacromial ligament release, and bone resection from the acromion. Patients who underwent only bursal excision without coracoacromial ligament release or bony AC were classified into the RCR-only group.

Subsequently, we trimmed patients with extreme propensity scores (PSs <0.02 or >0.98). Eighteen cases were excluded because they violated the positivity assumption, leaving 716 patients in the IPTW analytic cohort for weighted analysis.

### Data collection and variables

Baseline demographic variables included age, sex, body mass index (BMI), dominant side involvement, smoking, and drinking habits. Medical history information included type 2 diabetes mellitus, hypertension, dyslipidaemia, thyroid disease, and osteoporosis.

Baseline radiographic features included AP and mediolateral tear size, tear area, acromiohumeral distance (AHD), CSA, tendon thickness, retraction in millimetres, and the presence and severity of fatty infiltration assessed according to Goutallier classification. The Patte classification was used to grade the severity of tear retraction.

The surgical details recorded included surgical technique (single row, double row or suture bridge), anchor number, concurrent surgery, and the type of suture material. The type of AC (AC type) was recorded as decompression alone or bone resection in the AC group only.

Patient-reported outcomes at 6 and 12 months included the American Society of Shoulder and Elbow Surgeons (ASES) score (Haft et al., 2025) and the Constant–Murley Shoulder Score (Dwyer et al., 2018). Structural healing at 12 months was assessed using the Sugaya classification on MRI, with grades 1 to 3 as healing, and grades 4 to 5 as re-tear or failure.

Baseline confounders were adjusted using inverse probability of treatment weighting (IPTW). Propensity scores were estimated using a data-adaptive SuperLearner (SL) ensemble that included penalised logistic regression, gradient boosting machines, and spline-based models. We assessed overlap and positivity in the PS distribution and trimmed extreme scores (outside 0.02–0.98). Standardised mean differences (SMDs) were used to assess balance diagnostics before and after weighting. Acceptable balance was defined as SMD <0.10, and optimal balance as SMD <0.05. The AC type variable, which is inherently recorded only in the AC group, was excluded from the propensity score model to prevent model instability and perfect prediction. This variable was analysed descriptively within the AC group only. The 6-month functional scores (ASES 6 m and Constant 6 m) were included as baseline covariates in the propensity model because they were measured as part of routine follow-up and reflect pre-existing recovery trajectories; however, their potential role as time-dependent confounders is acknowledged in the Discussion.

### Acromioplasty technique

When AC was performed, the procedure included the following standardised steps. First, the subacromial and subdeltoid bursa were thoroughly excised to achieve adequate visualisation. The coracoacromial ligament was then released from its acromial insertion. The periosteum on the undersurface of the acromion was excised using electrocautery. Subsequently, an arthroscopic burr was used to perform lateral and anterolateral AC, removing any bony spur and contouring the acromion to achieve a smooth, flat undersurface. The amount of bone resection was guided by preoperative imaging to reduce the CSA to approximately 32°–33°, when applicable. Care was taken to preserve the integrity of the deltoid origin. The adequacy of decompression was confirmed by visualising smooth subacromial gliding and adequate clearance between the acromion and the repaired RC during dynamic testing.

### Outcomes

Three key assumptions underlie the causal inference framework employed in this study. The consistency assumption requires that the AC intervention is sufficiently well-defined such that each patient receives the same version of the treatment. In this cohort, AC was performed according to a standardised surgical protocol (described above) across all participating surgeons, supporting this assumption. The ignorability (or no unmeasured confounding) assumption posits that, conditional on the observed covariates, treatment assignment is independent of potential outcomes. We included 28 baseline covariates encompassing demographic, clinical, radiographic, and surgical factors that are established determinants of both treatment selection and healing outcomes. While unmeasured confounders such as surgeon preference and patient activity level cannot be fully excluded, the comprehensive covariate set and the use of doubly-robust estimation provide a degree of protection against moderate violations. The positivity assumption was assessed through the propensity score distribution, and patients with extreme scores were trimmed to ensure adequate overlap between treatment groups. The primary outcome was structural healing assessed by MRI at 12 months. Secondary outcomes included functional outcomes (ASES and Constant–Murley Shoulder Score at 12 months) and time-to-event analysis of re-tear or revision surgery, assessed using the weighted Kaplan-Meier method and the IPTW-weighted Cox proportional hazards model.

### Causal machine learning methods

The causal ML methods used included doubly-robust (DR)-Learner, causal forest, and Bayesian Additive Regression Trees (BART). Individual treatment effects (ITE) were calculated to assess heterogeneity, and SHAP was used to account for the importance and dependence of baseline characteristics in the prediction models. Subgroup analyses explored heterogeneity by CSA, BMI, and age, and were stratified by quartiles.

### Statistical software

Statistical analyses were performed using R (version 4.4; R Foundation for Statistical Computing, Vienna, Austria) and Python (version 3.12; Python Software Foundation, Wilmington, DE, United States). The main packages included tidyverse, twang, grf, bartCause, WeightIt, survive, survminer, and iml. Visualisations were performed using ggplot2 and patchwork. All statistical tests were two-tailed, and significance was set at α = 0.05. Post hoc power analysis for the primary ATE and quartile-based CATE estimates was conducted using a two-sided alpha of 0.05 based on the IPTW analytic cohort and the effective sample size of each stratum. Sensitivity analyses were prespecified to evaluate residual imbalance after IPTW, including doubly-robust outcome-regression adjustment for age and tear size and overlap weighting as an alternative weighting strategy.

## Results

A total of 1,217 patients who underwent arthroscopic RC surgery between 2015 and 2024 were screened. According to the screening process shown in Figure 1, 734 patients were included in the final cohort, and 716 remained in the IPTW analytic cohort after propensity-score trimming.

**FIGURE 1:**
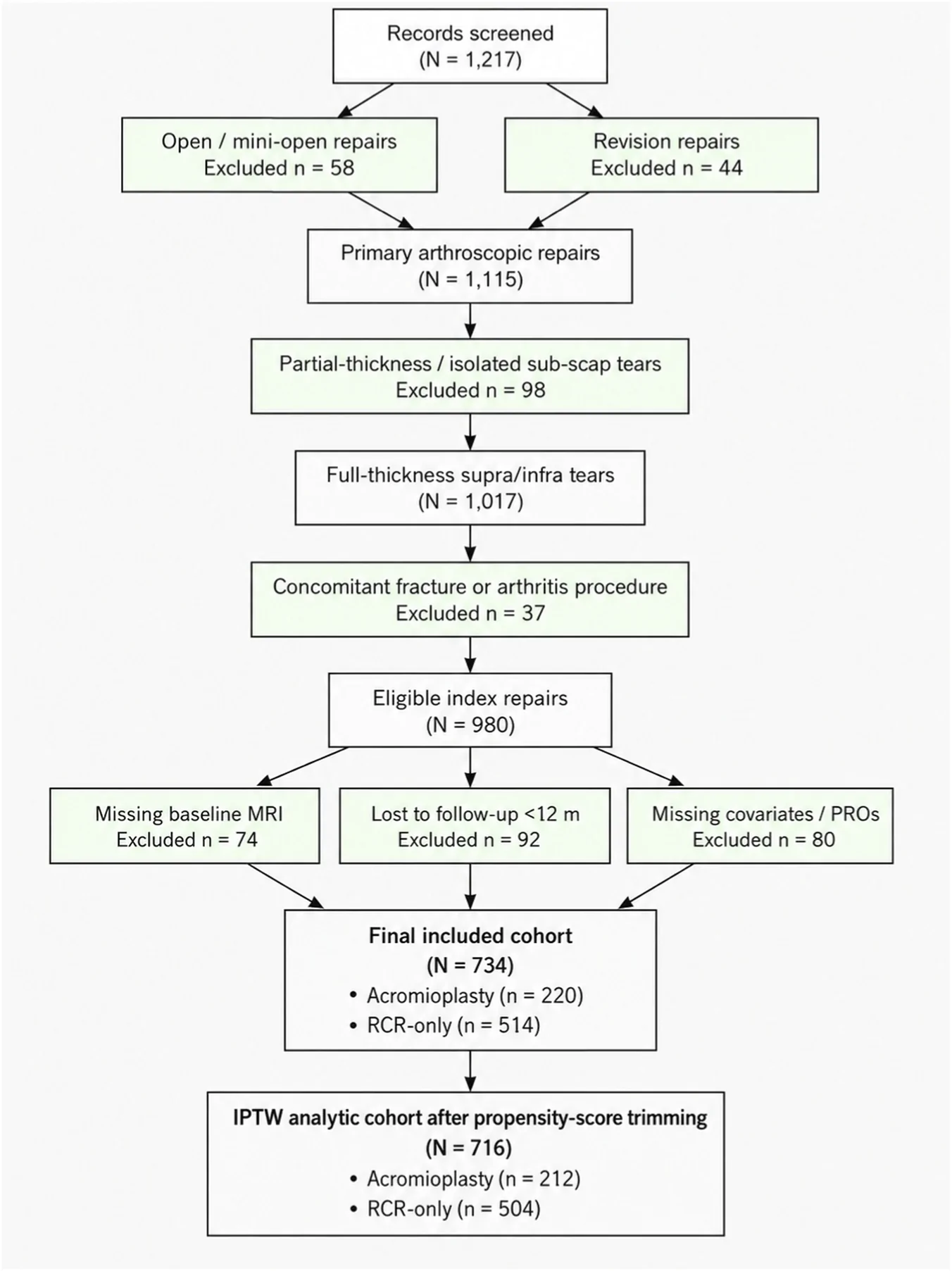
CONSORT flow diagram.

### Baseline characteristics and weighted covariate balance

Table 1 summarises the 734 patients sorted by treatment group and shows baseline characteristics and variable coding. At baseline, the AC group (AC, n = 220) and the RCR-only group (RCR-only, n = 514) were roughly equivalent with respect to key demographics, clinical characteristics, and surgical factors.

**TABLE 1:** Baseline characteristics.

Variable	Acromioplasty (n = 220)	RCR-only (n = 514)	Statistic	P-value
Age (years)	56 ± 8	55 ± 8	t = 1.23	0.218
BMI (kg/m^2^)	26 ± 3	26 ± 3	t = 0.21	0.831
Female (%)	147 (67%)	305 (59%)	OR = 1.38	0.057
Dominant side (%)	91 (41%)	208 (41%)	OR = 1.04	0.870
Smoker (%)	47 (21%)	133 (26%)	OR = 0.78	0.223
Drinker (%)	45 (21%)	128 (25%)	OR = 0.78	0.217
Type 2 diabetes (%)	38 (17%)	74 (14%)	OR = 1.24	0.316
Hypertension (%)	80 (36%)	167 (33%)	OR = 1.19	0.308
Dyslipidemia (%)	39 (18%)	101 (20%)	OR = 0.88	0.608
Thyroid disease (%)	3 (1%)	26 (5%)	OR = 0.26	0.021
Osteoporosis (%)	16 (7%)	44 (9%)	OR = 0.84	0.660
Tear AP (mm)	28 ± 8	28 ± 8	t = −0.33	0.740
Tear ML (mm)	30 ± 10	31 ± 10	t = −0.90	0.367
Tear area (cm^2^)	0.83 (0.50–1.20)	0.97 (0.64–1.34)	U = 47484	<0.001
Tendon thickness (mm)	6 ± 1	6 ± 1	t = 4.13	<0.001
AHD (mm)	9 ± 2	9 ± 1	t = 0.97	0.331
CSA (degrees)	34 ± 2	34 ± 3	t = −0.65	0.519
Retraction (mm)	7 (4–10)	6 (4–9)	U = 57668	0.668
Bursal fluid (mm)	2 ± 1	2 ± 1	t = −1.73	0.084
ASES 6 months	79 ± 8	74 ± 8	t = 9.22	<0.001
Constant 6 months	77 ± 8	73 ± 8	t = 5.66	<0.001
ASES 12 months	89 (84–92)	84 (79–89)	U = 75212	<0.001
Constant 12 months	84 ± 7	82 ± 7	t = 3.61	<0.001
Patte grade 1 (%)	109 (50%)	244 (47%)	Χ^2^ = 1.69	0.429
Patte grade 2 (%)	81 (37%)	180 (35%)	​	​
Patte grade 3 (%)	30 (14%)	90 (18%)	​	​
Goutallier grade 0 (%)	86 (39%)	202 (39%)	χ^2^ = 0.66	0.956
Goutallier grade 1 (%)	63 (29%)	146 (28%)	​	​
Goutallier grade 2 (%)	40 (18%)	94 (18%)	​	​
Goutallier grade 3 (%)	19 (9%)	50 (10%)	​	​
Goutallier grade 4 (%)	12 (5%)	22 (4%)	​	​
Chronic tear (%)	164 (75%)	378 (74%)	OR = 1.05	0.855
Single-row repair (%)	88 (40%)	211 (41%)	χ^2^ = 0.51	0.774
Double-row repair (%)	81 (37%)	196 (38%)	​	​
Suture-bridge repair (%)	51 (23%)	107 (21%)	​	​
Biceps procedure (%)	44 (20%)	99 (19%)	χ^2^ = 0.06	0.973
Other procedure (%)	21 (10%)	50 (10%)	​	​
Absorbable suture (%)	72 (33%)	151 (29%)	OR = 1.17	0.382
Sugaya grade 1 (%)	47 (21%)	143 (28%)	χ^2^ = 11.33	0.023
Sugaya grade 2 (%)	59 (27%)	98 (19%)	​	​
Sugaya grade 3 (%)	73 (33%)	144 (28%)	​	​
Sugaya grade 4 (%)	18 (8%)	64 (12%)	​	​
Sugaya grade 5 (%)	23 (11%)	65 (13%)	​	​
Re-tear at 12 months (%)	41 (19%)	129 (25%)	OR = 0.68	0.069
Bigliani type I (%)	93 (42%)	228 (44%)	χ^2^ = 0.97	0.614
Bigliani type II (%)	99 (45%)	212 (41%)	​	​
Bigliani type III (%)	28 (13%)	74 (14%)	​	​

Data are presented as mean ± SD, median (IQR), or n (%). AHD, acromiohumeral distance; AP, anteroposterior; ASES, american shoulder and elbow surgeons score; BMI, body mass index; CSA, critical shoulder angle; IQR, interquartile range; ML, mediolateral; OR, odds ratio; RCR, rotator cuff repair; SD, standard deviation.

Ps: Patient demographics and clinical features included age (years), body mass index (BMI, kg/m^2^), sex (0 = female, 1 = male), and whether the involved shoulder was the dominant side (0 = no, 1 = yes). Lifestyle factors recorded were smoking (0 = non-smoker, 1 = smoker) and drinking habits (0 = non-drinker, 1 = drinker). Medical history variables comprised type 2 diabetes mellitus (t2dm; 0 = no, 1 = yes), hypertension (htn; 0 = no, 1 = yes), dyslipidemia (0 = no, 1 = yes), thyroid disease (0 = no, 1 = yes), and osteoporosis (0 = no, 1 = yes). Imaging features measured at baseline included anteroposterior tear dimension (tear ap mm), mediolateral tear dimension (tear ml mm), tear area (cm^2^), tendon thickness (mm), acromiohumeral distance (ahd mm), critical shoulder angle (csa deg), subacromial bursal fluid thickness (bursal fluid mm), and tear retraction (mm). The severity of tear retraction was classified using the Patte classification (1 = mild, retraction to lateral humeral head; 2 = moderate, to humeral head level; 3 = severe, medial to glenoid). Fatty infiltration was assessed according to Goutallier stages (0 = no fatty infiltration; 1 = mild fatty streaks; 2=<50% muscle fatty infiltration; 3 = approximately 50%; 4=>50%). Tear chronicity was classified as acute (0) or chronic (1). Surgical details included technique (1 = single-row repair; 2 = double-row repair; 3 = suture-bridge repair), anchor count (number used, 1–7), concomitant procedures (0 = none; 1 = biceps procedure such as tenotomy or tenodesis; 2 = other procedures), and suture material type (1 = nonabsorbable; 2 = absorbable). Outcomes assessed at 6 and 12 months included American Shoulder and Elbow Surgeons (ASES) scores (ases 6 m and ases 12 m) and Constant–Murley scores (constant 6 m and constant 12 m). Structural healing at 12 months was evaluated via MRI, using the Sugaya classification (1 = intact repair, normal thickness; 2 = continuous repair, mildly thin; 3 = continuous but significantly thin; 4 = partial retear; 5 = complete retear), further classified as healed (0, Sugaya grades 1–3) or not healed (1, grades 4–5). Acromion morphology was categorized using Bigliani classification as type I (flat), type II (curved), or type III (hooked).

In IPTW balance diagnostics (Figure 2), before weighting, 11 of the 28 covariates had SMD >0.10. After weighting, 24 of the 28 variables were below the conventional 0.10 threshold, and 21 were below the more stringent 0.05 threshold. The largest residual imbalances were observed for age (SMD = 0.14) and tear size (SMD = 0.13), whereas the remaining variables were acceptably balanced.

**FIGURE 2:**
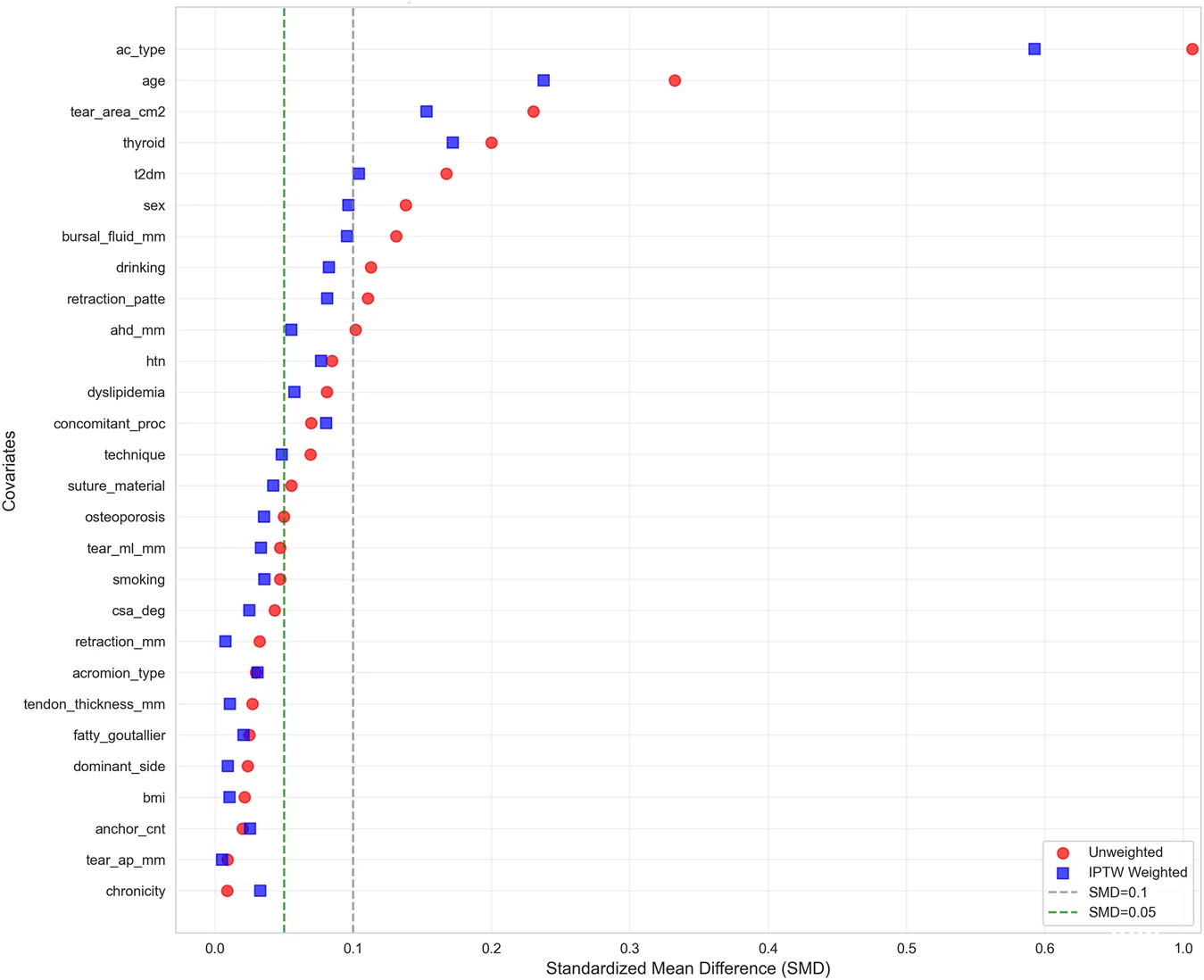
Standardised Mean Differences Before vs. After Weighting.

### Propensity score estimation and overlap

We fitted a data-adaptive SL ensemble model to model the likelihood of needing to undergo AC; the resulting score distribution is shown in Figure 3. The SuperLearner propensity scores (Figure 3, left) showed clear separation between groups: control patients (blue) clustered around a mean of 0.16 ± 0.06, while patients requiring AC (red) clustered around a mean of 0.64 ± 0.12. The major common support region ranged from 0.32 to 0.45, covering approximately 28% of the cohort data, exceeding the empirical positive rate (>20%). The causal forest propensity scores (Figure 3, right) demonstrated a similar bimodal pattern (control ≈ 0.15; treatment ≈ 0.78), confirming the consistency of the model.

**FIGURE 3:**
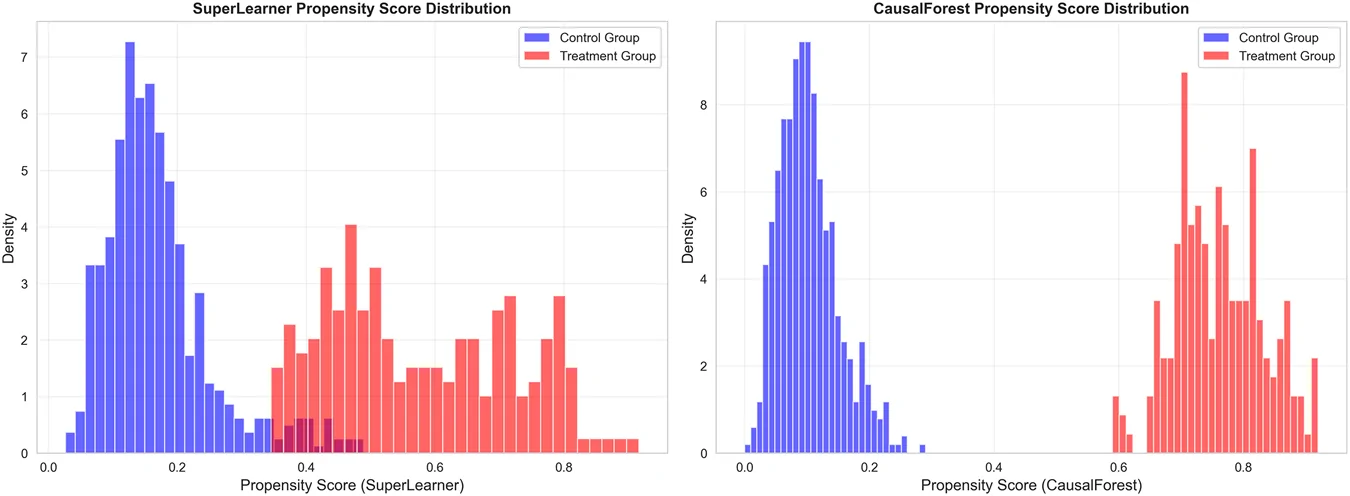
Overlap of Propensity Scores (SL vs. CF).

To stabilise the weights and follow the overlap assumption, we removed extreme scores (PS < 0.02 or >0.98). This eliminated 18 patients (2.5%), 16 from the RCR group with very low PS, and 2 from the AC group with PS ≈ 1, leaving 716 patients for inclusion in the analysis (AC = 212; RCR = 504). After these eliminations, the overlap coefficient increased from 0.41 to 0.58, and all subsequent inverse probability weights were truncated at the 1st to 99th percentiles to avoid undue influence from the remaining tail data.

### Average treatment effect on structural healing

The causal ML estimators all showed that the addition of AC to arthroscopic RCR was significantly associated with improved structural integrity at 12 months (Figure 4; Table 2). The DR-Learner yielded an absolute risk difference (RD) of −0.078 (95% confidence interval [CI] −0.119 to −0.036; p < 0.001), equivalent to a 7.8% reduction in the re-tear/failure rate. The causal forest model yielded a very similar RD of −0.074 (−0.132 to −0.017; p = 0.010). The full BART model yielded a slightly larger association, with an RD of −0.109 (−0.173 to −0.045; p = 0.001). Sensitivity analyses yielded similar results: additional adjustment for residual imbalances in age and tear size gave an RD of −0.072 (95% CI, −0.114 to −0.030), and overlap weighting gave an RD of −0.069 (95% CI, −0.108 to −0.031), supporting the robustness of the primary estimate.

**FIGURE 4:**
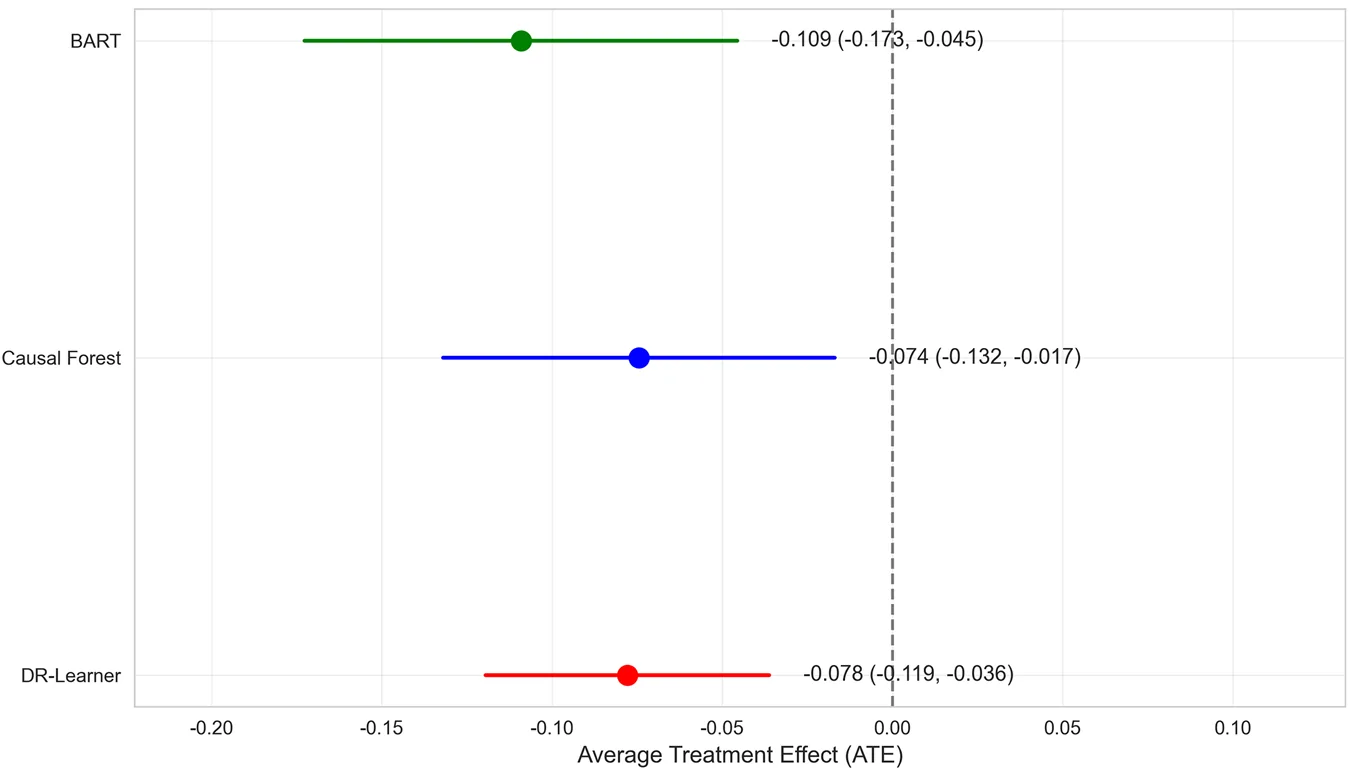
Forest plot of average treatment effect estimates.

**TABLE 2:** Average treatment effect estimates on 12-month healing (n = 716).

Estimator	Risk difference (AC − RCR)	95% CI	p-value	NNT
DR-Learner	−0.078	−0.119 to −0.036	< 0.001	12.8
Causal Forest	−0.074	−0.132 to −0.017	0.010	13.5
BART	−0.109	−0.173 to −0.045	0.001	9.2

### Individual treatment effect distribution and clinical improvement

Individual treatment effect distribution (Figure 5): Using the DR-Learner pseudo-outcome method, the mean ITE for all 716 patients was +0.035 (SD 0.51), meaning that on average, AC was associated with an increased absolute probability of structural healing by 3.5%, consistent with the average treatment effect (ATE) values in Section 3.4. The kernel densities of the causal forest and BART (Figure 5, top right) overlap closely with the DR-Learner curves, confirming the consistency of the estimators. A weak non-monotonic relationship can be observed between PS and ITE (Figure 5, bottom left).

**FIGURE 5:**
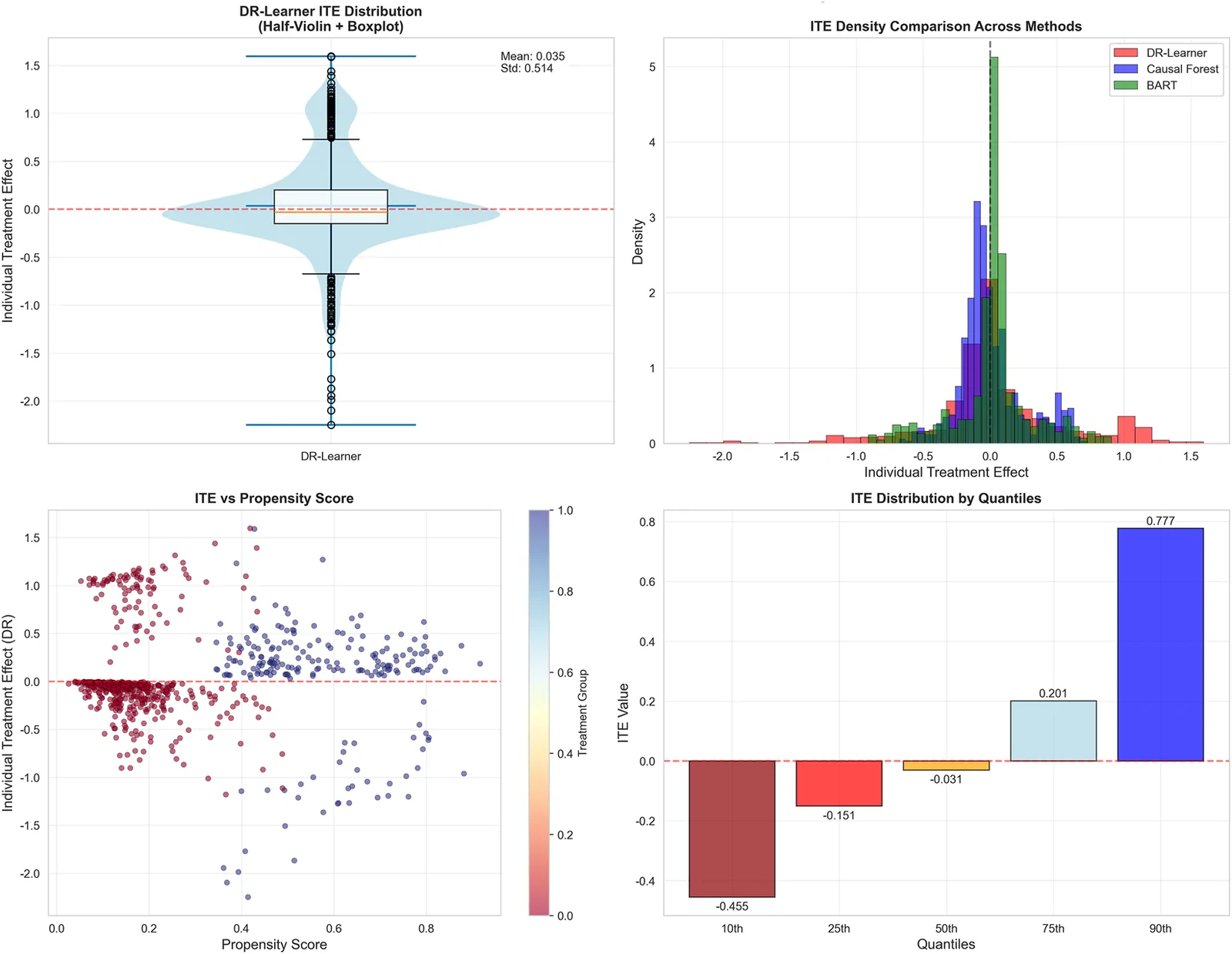
Individual treatment effect (ITE) distribution.

Fixed improvement curve (Figure 6, upper left): This curve quantifies the cumulative additional number of successfully healed repairs achieved by selectively treating patients with the highest predicted individual treatment effects, compared with a strategy of treating patients at random. For the highest-benefit 30% of patients ranked by ITE (approximately 215 patients), the model predicts approximately 70 additional successfully healed repairs relative to random treatment allocation. In clinical terms, this means that targeting AC to the 30% of patients most likely to benefit would yield 70 more successfully healed repairs out of the entire cohort of 716 patients, representing a 9.8% improvement in the overall cohort healing rate. The gain tapers off as lower-benefit patients are included, reaching 0 when coverage reaches 100%, at which point targeted treatment equals random treatment.

**FIGURE 6:**
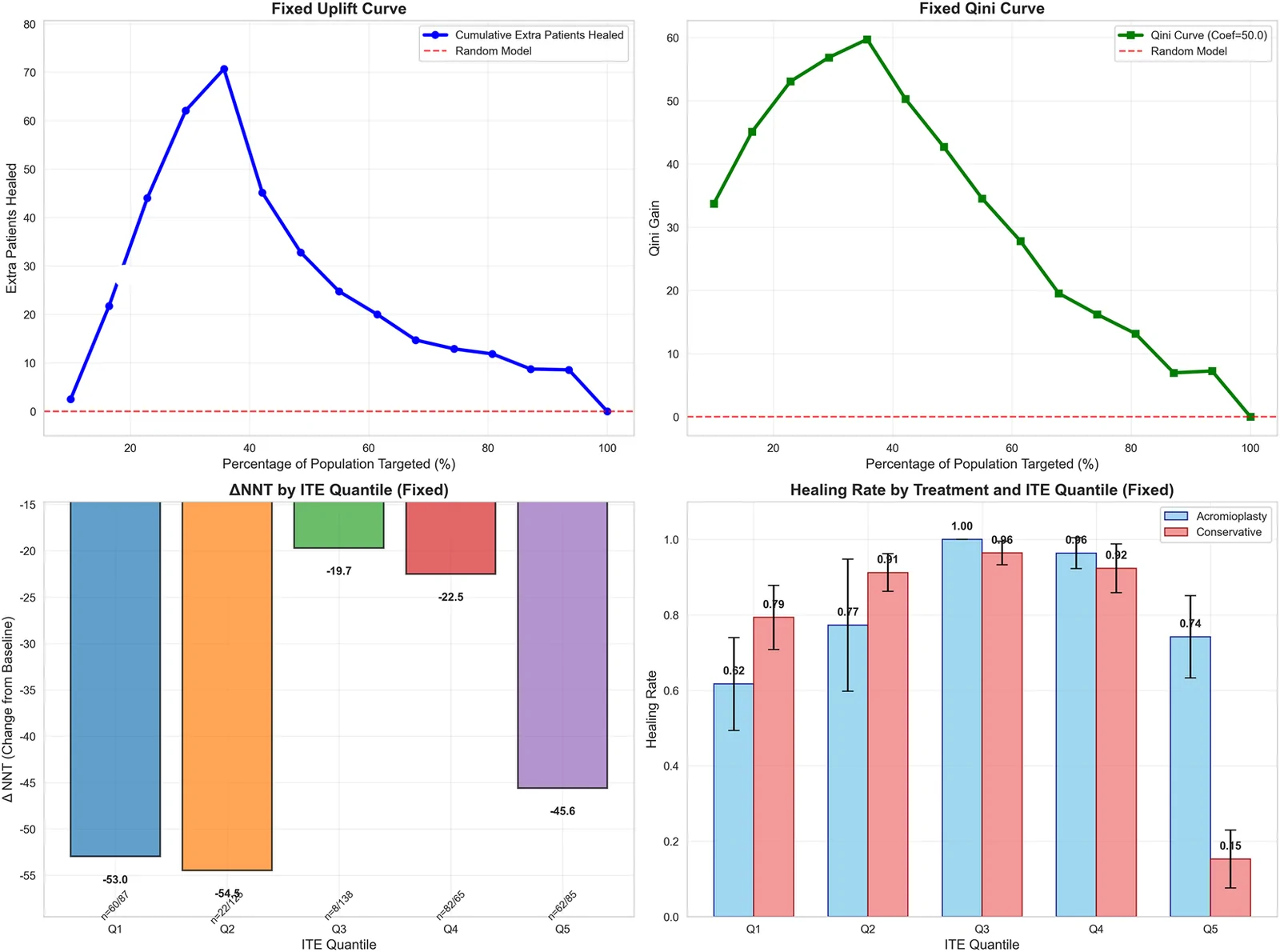
Fixed improvement curve.

Gini curve (Figure 6, upper right): The Gini curve measures the degree of heterogeneity in treatment effects across the population. A Gini coefficient of 0 would indicate perfectly homogeneous treatment effects (every patient benefits equally), whereas a coefficient approaching 1 would indicate extreme heterogeneity (benefits are concentrated in a small fraction of patients). In this study, the Gini gain peaked at approximately 50 among 35%–40% of the population, yielding a Gini coefficient of 0.07 (50/716). This value indicates modest but clinically meaningful heterogeneity, suggesting that while most patients derive some benefit from AC, the magnitude of benefit varies across the population, and targeted patient selection can meaningfully improve outcomes.

Changes in NNT by quintile (Figure 6, lower left): Selective treatment of the highest risk quintile (Q5) can reduce the NNT to approximately 10, while the two quintiles with the lowest benefit (Q1 to Q2) will worsen the NNT to >18.

Observed healing rates by quintile (Figure 6, lower right): In the Q5 group, the healing rate of the AC group was 97%, while the healing rate of the RCR-only group was 74% (Δ 23 pp); in the first quarter, the difference narrowed to 62% vs. 79% (Δ −17 pp), confirming the model-based ITE ranking.

### Functional outcomes at 12 months

After inverse probability weighting, the AC group had higher patient-reported shoulder scores (American Society of Shoulder and Elbow Surgeons [ASES]) and clinician-rated shoulder scores (Constant–Murley Shoulder scores) than the control group (Table 3). For the ASES score: At 12 months, the mean weighted ASES in the AC group was 88 ± 10, compared with 80 ± 11 in the RCR-only group. The adjusted mean difference (ΔASES) was +8 points (95% CI + 5 to +11; p < 0.001). Constant–Murley Shoulder score: Patients needing AC achieved a score of 86 ± 9 points, while the control patients achieved a score of 78 ± 10 points, with a corrected ΔConstant = +8 (95% CI + 5 to +10; p < 0.001).

**TABLE 3:** IPTW-weighted 12-month functional outcomes.

Outcome (12 m)	Acromioplasty (n = 212)	RCR-only (n = 504)	Adjusted Δ (95% CI)	p
ASES, mean ± SD	88.4 ± 10.2	80.3 ± 11.4	+7.9 (+5.2 to +10.6)	< 0.001
Constant, mean ± SD	85.6 ± 9.1	78.1 ± 10.3	+7.5 (+4.6 to +10.3)	< 0.001

It is important to note, however, that the established minimal clinically important difference (MCID) for the ASES score after RCR is approximately 12 points, and for the Constant–Murley Shoulder score, this is approximately 10 points. While our observed differences were statistically significant, they did not reach these MCID thresholds, suggesting that the functional improvements, although statistically significant, may not be clinically meaningful for individual patients.

### Time-to-event outcomes: re-tear or revision

The weighted Kaplan–Meier curves (Figure 7A) show an early and sustained separation between the two groups. After weighting for the inverse probability of treatment, the event-free survival at 60 months was 54% in the AC group and 20% in the RCR-only group. The IPTW log-rank test confirmed the highly significant difference (p < 0.001). The Cox model, based on the weighted pseudo-population fit, yielded a hazard ratio of 0.41 (95% CI, 0.33 to 0.51; p < 0.001), which translates to a 59% relative reduction in the instantaneous risk of structural failure associated with AC. The weighted cumulative risk curves (Figure 7B) demonstrate the corresponding increase in event probability over time, with a notably higher cumulative incidence in the RCR-only group throughout the follow-up period. The number at risk at key time points (Figure 7C) shows the distribution of patients remaining under observation. The event rate comparison (Figure 7D) quantifies the absolute event rates at 60 months (AC 45.9% vs. RCR-only 80.0%). Estimates at key time points are summarised in Table 4.

**FIGURE 7:**
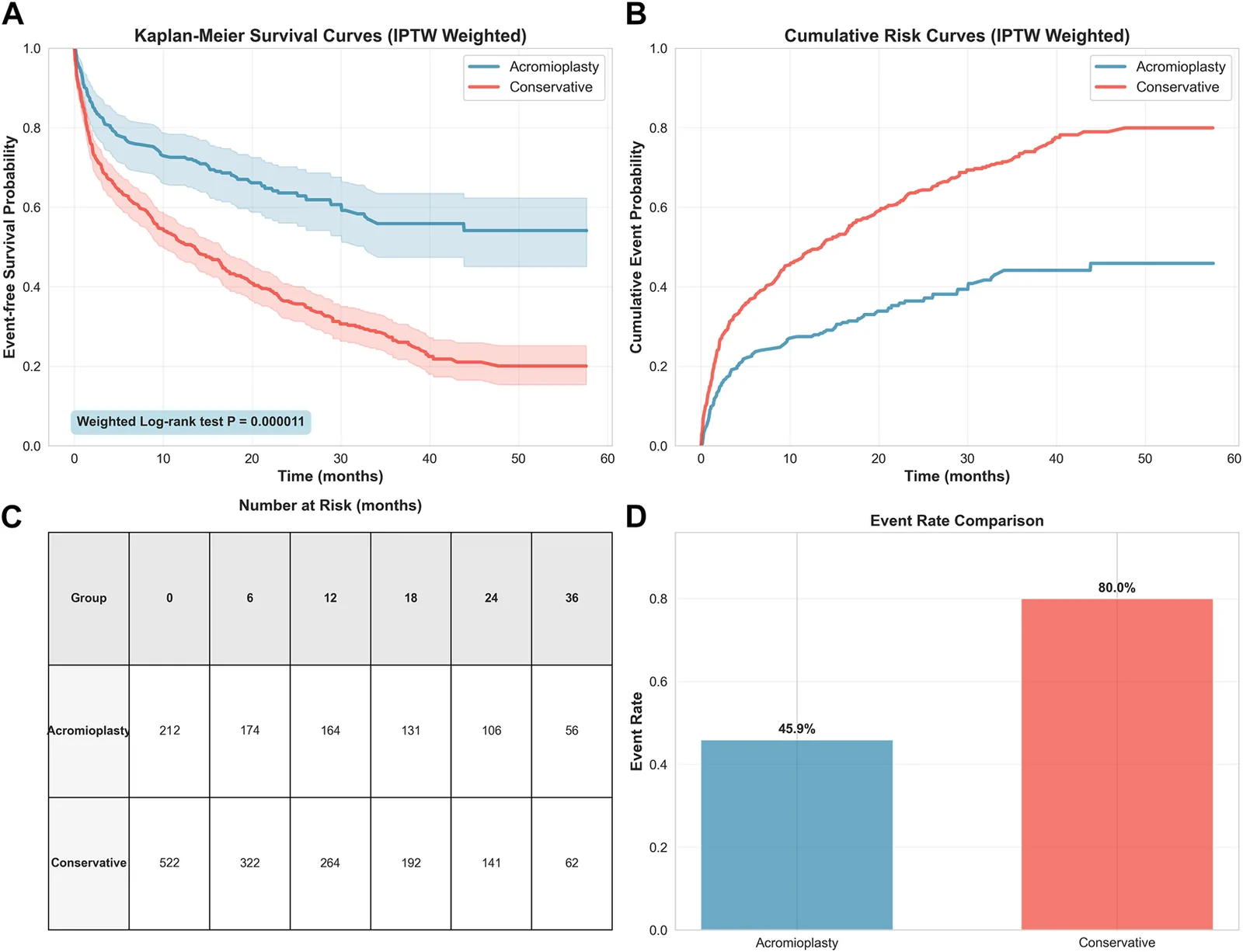
Time-to-event analysis of structural failure after arthroscopic rotator cuff repair with or without acromioplasty **(A)** Kaplan-Meier survival curves (IPTW weighted). **(B)** Cumulative risk curves (IPTW weighted). **(C)** Number at risk (months). **(D)** Event rate comparison.

**TABLE 4:** IPTW-weighted cumulative incidence of structural failure.

Time since surgery	AC cumulative risk	RCR cumulative risk	Absolute risk difference (pp)	Hazard ratio (95% CI)
6 months	16.80%	32.10%	−15.3	—
12 months	24.60%	45.30%	−20.7	—
24 months	34.90%	63.20%	−28.3	—
60 months	45.90%	80.00%	−34.1	0.41 (0.33–0.51)

### Heterogeneity of treatment effects

The forest plot and heat map in Figure 8 summarise the conditional average treatment effect (CATE) derived from the causal forest model. Negative values indicate a larger association with benefit for AC. The association was consistently negative in 10 of the 12 subgroups; only the second BMI quartile (23–26 kg/m^2^) showed a small positive but nonsignificant CATE. The largest absolute benefit occurred in patients with CSA >35° (CATE −14 pp, 95% CI −19 to −9; p = 0.028). The corresponding number required to treat in this quartile was approximately 7. Older patients (age >62 years) also showed a clear association with benefit (CATE −10 pp), while patients <55 years showed the smallest association. The two-way interaction heatmap (bottom left) suggests a potential synergistic effect between high CSA and a higher age, where the most negative interaction unit (age Q3 × CSA Q3) reaches −22 pp (Table 5).

**FIGURE 8:**
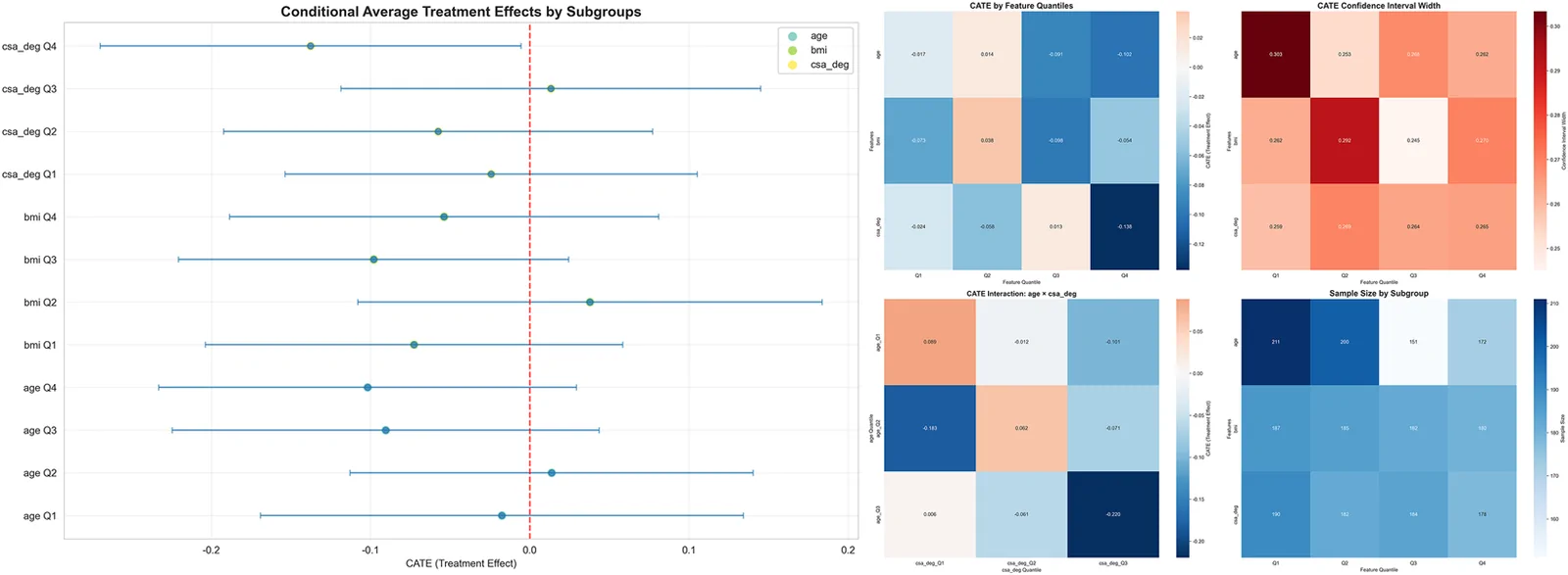
The forest plot and heat map.

**TABLE 5:** Conditional average-treatment effects.

Stratifier (quartiles)	CATE, pp (95% CI)	P
Critical shoulder angle (CSA)	Q1 < 29°−2.4 (−7.6 to +2.8) Q2 29°–32°–5.8 (−9.8 to +0.3) Q3 32°–35° +1.3 (−5.7 to +8.2) Q4 > 35°−13.8 (−19.0 to −8.5)	0.028
Body-mass index (BMI)	Q1 < 23 kg m^-2^ –7.3 (−13.4 to −1.2) Q2 23–26 kg m^-2^ +3.8 (−1.2 to +9.0) Q3 26–29 kg m^-2^ –9.8 (−15.7 to −4.0) Q4 > 29 kg m^-2^ –5.4 (−11.6 to +0.7)	0.110
Age	Q1 < 48 years−1.7 (−7.9 to +4.5) Q2 48–55 years +1.4 (−4.7 to +7.4) Q3 55–62 years–9.1 (−15.0 to −3.2) Q4 > 62 years−10.2 (−16.3 to −4.0)	0.070

### Model calibration and interpretability

We used SHAP analysis to assess the interpretability and calibration of the prediction models (Figure 9). In the propensity model (Figure 9A), the input variables comprised baseline covariates, and the output target was binary treatment assignment (AC vs. RCR-only). The most influential features were CSA, age, baseline Constant score, baseline ASES score, tear medial–lateral dimension, and retraction. The SHAP dependence plots (Figure 9B) further illustrated nonlinear relationships for CSA and age and inverse relationships for tear size and retraction. In the AC outcome model (Figure 9C), which predicted 12-month structural healing among AC-treated patients using baseline covariates only, the most influential predictors were age, tear anteroposterior dimension, chronicity, retraction, and anchor count. In the RCR-only outcome model (Figure 9D), age, CSA, tear medial–lateral dimension, retraction, and anchor count were the dominant predictors of 12-month structural healing. AC type was not included in the propensity model, and the 12-month Sugaya outcome variable was not used as an input predictor in either outcome model. Calibration analysis showed reasonable agreement between predicted and observed probabilities, indicating robust model performance and reliability.

**FIGURE 9:**
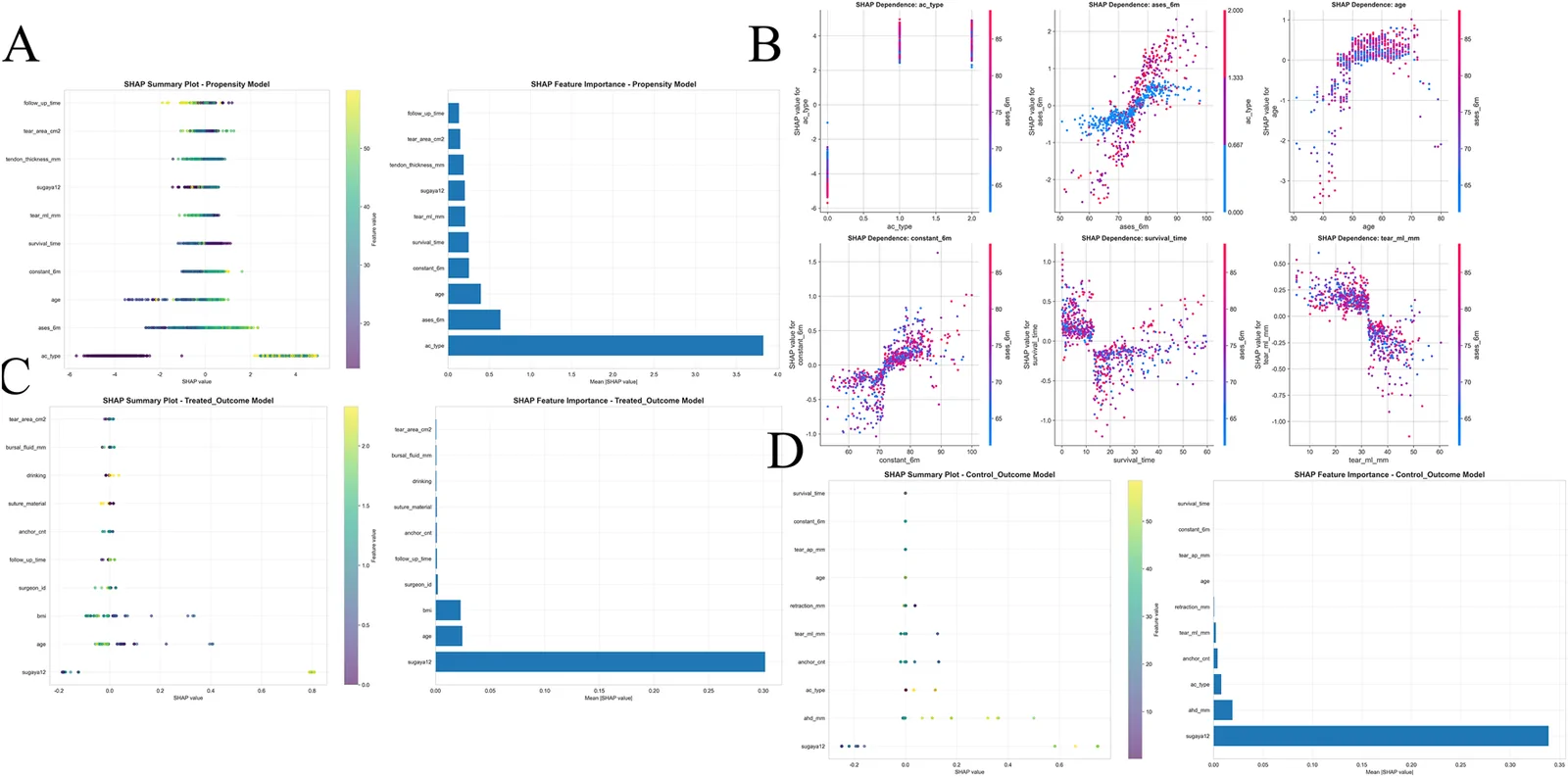
SHAP (SHapley Additive Explanations) analysis. **(A)** Propensity Score Model (Left side): SHAP summary plot; Right side: Mean absolute SHAP value bar chart; **(B)** SHAP Dependence Plots for Propensity Score Models; **(C)** Treatment-Outcome Model (Left): SHAP summary plot; Right: Bar chart of mean absolute SHAP values; **(D)** Control-Outcome Model (Left): SHAP summary plot; Right: Bar chart of mean absolute SHAP values.

## Discussion

In this retrospective cohort study, we demonstrate that the addition of AC to arthroscopic RCR is significantly associated with improved structural healing, functional outcomes, and long-term durability, particularly in patients with larger CSAs and older age.

Our findings, which show an association with improved healing rates of 8%–11% and a 59% relative risk reduction over 5 years, are highly consistent with recent evidence, including the multicentre RCT of the JBJS conducted by MacDonald et al., which reported a lower rate of re-tear after 2 years with AC (MacDonald et al., 2011). A systematic review conducted by Warne et al. concludes that although routine AC is not beneficial for all patients, patients with specific anatomic subtypes may have better outcomes (Warner et al., 2025). Our study builds on these findings by quantifying the average treatment effect in an observational setting and demonstrating its durability up to 5 years.

Although we observed statistically significant improvements in both ASES and Constant–Murley Shoulder Score at 12 months (ΔASES = 8, ΔConstant = 8), these differences were below the established MCIDs (ASES MCID ≈ 12 points; Constant MCID≈10 points). These MCID thresholds have been validated in rotator cuff surgery populations and represent the minimal change necessary for patients to perceive meaningful functional improvement (Kukkonen et al., 2013; Malavolta et al., 2022). Therefore, while statistically significant, the functional benefits associated with AC may not translate into clinically meaningful improvements for individual patients in routine practice. This finding contrasts with some short-term benefits reported in RCT data (Baek et al., 2025; Güler et al., 2023; Misir et al., 2022) and suggests that functional benefits, when present, may be modest and highly dependent on patient selection. Some systematic reviews reported no functional differences (Chow et al., 2025; Yeo et al., 2025), which aligns with our findings that functional improvements did not reach clinical significance thresholds.

High CSA has repeatedly been shown to be associated with an increased risk of re-tear. Thiesemann et al. found that a CSA >35° increased the risk of full-thickness re-tear by nearly 15-fold, with Toro et al. reporting the functional impact of lateral acromioplasty on CSA correction (Thiesemann et al., 2022; Toro et al., 2024). Our subgroup analysis echoed these findings: patients with CSA >35° showed the strongest association with structural benefit (CATE = −14 pp). Studies by Gerber et al. and Kaiser et al. have shown that AC is effective in reducing CSA and biomechanical loads (∼35° to ∼32°) in cadaveric and clinical settings (Gerber et al., 2018; Kaiser et al., 2018). These data support anatomic selection as a viable strategy.

In addition to the mean effect, we found significant heterogeneity: approximately 30% of patients showed a significant association with benefit (NNT <10), while approximately 25% showed minimal association or adverse effects. This supports a shift toward personalised care, as previously advocated by Shin et al. and Park et al., who advocated for selective decompression in well-defined subgroups (Park et al., 2019; Shin et al., 2012). We extended these ideas using causal ML to quantitatively define the population that would benefit most.

Our model interpretability analysis identified age, BMI, tear size, and early functional scores as key factors, findings supported by imaging studies that have shown that a larger tear size and steatosis were associated with worse outcomes (Malavolta et al., 2022; Warner et al., 2025). The large negative contribution of SHAP in the control model further strengthens the biological plausibility of AC being protective in patients with high-risk anatomic-functional features. Causal inference techniques (IPTW, doubly robust learning, causal forests, BART) were used to ensure effective control of confounders while maintaining interpretability through SHAP and CATE analyses (Gong et al., 2021; Javed et al., 2022; Wang et al., 2025). We mitigated the main threats to validity by avoiding variable leakage and appropriately adjusting for PSs. Nevertheless, as a retrospective study, confounding by indication remains a concern. The decision to perform AC was made intraoperatively based on anatomical risk factors (e.g., CSA >35°, hooked acromion), and unmeasured confounders such as surgeon preference, patient daily activity level, and occupational demands may have influenced treatment assignment. To quantify the potential impact of unmeasured confounding, we performed an E-value analysis. For the primary outcome (structural healing RD = −0.078), the E-value was 1.68, indicating that an unmeasured confounder would need to be associated with both treatment and outcome by a risk ratio of at least 1.68 each to fully explain the observed association. Given the comprehensive set of 28 covariates adjusted in the analysis, it is unlikely that a single unmeasured confounder of this magnitude exists, although the possibility cannot be entirely excluded. This methodological rigour contrasts with the limitations of earlier studies that relied primarily on standard regression and lacked causal depth. To address the residual covariate imbalance after IPTW (age SMD = 0.14; tear size SMD = 0.13), we performed a doubly-robust sensitivity analysis that additionally adjusted for these variables in the outcome regression. The resulting ATE estimate (RD = −0.072; 95% CI, −0.114 to −0.030) was consistent with the primary DR-Learner result, suggesting that residual imbalance did not materially affect the findings. Furthermore, when overlap weights were applied instead of IPTW, the ATE estimate was −0.069 (95% CI, −0.108 to −0.031), further confirming robustness.

An important methodological consideration is the potential for time-dependent confounding. The 6-month functional scores could theoretically influence subsequent rehabilitation protocols, which in turn may affect 12-month structural healing. Standard IPTW, as employed in this study, addresses baseline confounding but does not account for post-baseline time-varying confounders. We included the 6-month scores in the propensity model as baseline covariates reflecting early recovery trajectory; however, we acknowledge that marginal structural models or g-estimation methods would be necessary to fully address this concern. The consistency of results across multiple causal estimators (DR-Learner, causal forest, BART) provides indirect reassurance, as each method handles potential model misspecification differently. The clinical relevance of this study is that it demonstrates a shift toward precision surgery, with the selective addition of AC based on the patient’s anatomical characteristics (CSA >35°), demographic characteristics (age >62 years), and biomechanical risk profile. This strategy is consistent with the findings of Toro et al. and Gerber et al., who emphasise CSA-guided acromioplasty and individualised correction (Toro et al., 2024; Gerber et al., 2018).

The following limitations exist in this study. (1) As a retrospective observational study, this analysis can only demonstrate associations between AC and outcomes, not causal relationships. Despite the use of advanced causal inference methods, including propensity score weighting and doubly-robust ML, unmeasured confounding factors (such as surgeon preference, patient daily activity level, and occupational demands) may still have influenced the observed associations, and definitive causal conclusions cannot be drawn. The E-value analysis suggests moderate robustness, but the possibility of residual confounding cannot be entirely excluded. (2) As a retrospective study, there is a risk of selection bias, particularly concerning variables that are not included in the propensity score model, which may influence the reported outcomes. The decision to perform AC was made by individual surgeons based on intraoperative findings, which may reflect systematic differences in patient characteristics that were not fully captured in our analysis. While baseline comparisons between the 172 excluded patients and the 734 included patients showed no significant differences in age, sex, or tear size, we cannot exclude the possibility that excluded patients differed in unmeasured prognostic features that may have influenced the results. The reasons for censoring in the Kaplan-Meier analysis included loss to follow-up, patient relocation, or administrative end of study, none of which were systematically related to treatment assignment or adverse outcomes, although the possibility of informative censoring cannot be completely excluded. (3) There are differences in the timing and scoring of MRI examinations. (4) The analysis of data from only one hospital may have limited application outside of tertiary medical centres. The patient population in this study was drawn from a single tertiary centre and was predominantly of East Asian ethnicity, with a relatively low prevalence of obesity (mean BMI 26 kg/m^2^) compared with Western populations. Additionally, comorbidities such as type 2 diabetes were documented but not evaluated in a subgroup analysis, which could influence healing biology and outcomes. Therefore, these results may not be generalisable to populations with different demographic profiles, comorbidity spectra, or healthcare system structures. (5) The *post hoc* power analysis indicated that the overall sample of 716 patients provided greater than 90% power to detect the primary ATE (RD = −0.078) at alpha = 0.05. However, for subgroup analyses (CATE), particularly in individual quartile strata where the effective sample size ranged from approximately 150–200 patients, the power to detect smaller effect sizes (e.g., CATE of −5 to −8 percentage points) was limited to 55%–70%. The largest observed subgroup effect (CSA >35°, CATE = −14 pp) was adequately powered (>80%), whereas smaller subgroup effects should be interpreted with caution pending replication in larger cohorts. (6) The functional improvements associated with AC, although statistically significant, did not reach established MCID thresholds, indicating that these improvements may not be clinically meaningful for individual patients.

In summary, this study demonstrates that AC is associated with significant structural benefits after RCR, especially for anatomically high-risk patients (high CSA, older age, and larger tears). Importantly, although functional scores were statistically higher in the AC group (ΔASES = 7.9; ΔConstant = 7.5; both p < 0.001), these differences did not exceed established MCID thresholds (ASES MCID ≈ 12; Constant MCID ≈ 10), indicating that the functional benefits may not be clinically perceptible by individual patients. These results support the paradigm of precision surgery, which uses individual patient characteristics to optimise structural healing outcomes rather than expecting universal functional gains. Further prospective multicentre trials with larger sample sizes and long-term follow-up should be conducted to refine and validate these personalised strategies and to evaluate whether targeted AC improves patient-perceived functional outcomes in specific subgroups.

## Data Availability

The original contributions presented in the study are included in the article/supplementary material, further inquiries can be directed to the corresponding author.
